# Development of luciferase-linked antibody capture assay based on luciferase immunoprecipitation systems for antibody detection of porcine reproductive and respiratory syndrome virus

**DOI:** 10.1186/s12896-018-0483-5

**Published:** 2018-11-16

**Authors:** Jie Li, Gang Wang, Di Yang, Bao Zhao, Yongpan Zhao, Yonggang Liu, Xuehui Cai, Yuchen Nan, En-Min Zhou, Chunyan Wu

**Affiliations:** 1 Department of Preventive Veterinary Medicine, College of Veterinary Medicine, Northwest A&F University, Yangling, 712100 Shaanxi China; 2 Scientific Observing and Experimental Station of Veterinary Pharmacology and Diagnostic Technology, Ministry of Agriculture, Yangling, 712100 Shaanxi China; 3 State Key Laboratory of Veterinary Biotechnology, Harbin Veterinary Research Institute of Chinese Academy of Agricultural Sciences, Harbin, 150001 China; 4 Shaanxi Animal Disease Control Center, Xi’an, 710016 Shaanxi China; 5 Shaanxi Domestic Animal Improving Station, Xianyang, 713702 Shaanxi China

**Keywords:** PRRSV, Luciferase immunoprecipitation systems, luciferase-linked antibody capture assay, Antibody detection, ELISA

## Abstract

**Background:**

Early detection of porcine reproductive and respiratory syndrome virus (PRRSV) infection of swine is necessary to control this devastating disease. By monitoring host serum antibodies to viral antigens, early virus detection within herds is feasible. In this study, recombinant antigens were generated using recombinant DNA techniques to fuse PRRSV structural protein (N) or nonstructural protein 1α (nsp1α) with the *Rellina* luciferase gene. Next, fused genes were cloned into plasmids and transfected into HEK-293 T cells for transient expression. Upon co-incubation of lysates with pig sera, antigen-antibody complexes formed that bound to Protein-G coated onto microplates. By further measurement of luminance value, a modified form of Luciferase Immunoprecipitation Systems, namely luciferase-linked antibody capture assay (LACA) was developed for detection of PRRSV-specific antibodies.

**Results:**

Known anti-PRRSV antibody-positive or -negative serum samples (125 and 122 samples, respectively) were used to validate the LACA and compared it with IDEXX PRRS ×3 ELISA. Based on the result, N-*Rluc* and nsp1α-*Rluc* LACA results were 95.3 and 94.4% in agreement with IDEXX ELISA, suggested a similar specificity of LACA to IDEXX ELISA. Moreover, when both LACA and IDEXX ELISA were used to evaluate sequential serum samples obtained from PRRSV experimentally infected pigs, the PRRSV-specific antibody response was detectable as early as 3 days post-inoculation (dpi) using N-*Rluc* LACA, but undetectable until 7 dpi using IDEXX ELISA, suggesting an improved sensitivity of LACA. Meanwhile, antibodies specific for nsp1α were detected at higher levels overall, but were undetectable until 10 dpi. Furthermore,. Notably, one IDEXX ELISA positive result was not confirmed by LACA or IFA and was thus considered a false-positive result.

**Conclusion:**

The LACA exhibited similar specificity but improved sensitivity to that of the commercial IDEXX PRRS ×3 ELISA kit for detection of PRRSV-specific antibodies in pig serum. Importantly, LACA could be adapted for detecting antibodies against other PRRSV targets, such as nsp1α, to achieve earlier detection of PRRSV infection.

## Background

Porcine reproductive and respiratory syndrome virus (PRRSV) is a positive-stranded enveloped RNA virus which belongs to the genus *Arterivirus*, family *Arteriviridae* and order *Nidovirales* [1]. Recently, a new proposal has classified PRRSV isolates into two species in the genus *Porartevirus*, *PRRSV-1* and *PRRSV-2,* that replace their previous designations of European-like and North American-like genotypes, respectively [2, 3].

The antibody response to PRRSV infection is highly complicated and is still not fully understood. However, multiple methods have been established to detect PRRSV specific antibody as a serological marker for PRRSV infection, such as enzyme-linked immunosorbent assay (ELISA), immunoperoxidase monolayer assay (IPMA) and immunofluorescence and immunochromatographic strip-based assays [4–9]. Generally, PRRSV-specific Neutralizing antibodies (NAbs) appear typically after 28 days post-inoculation (dpi) [1, 10], but non-protective antibodies produced within the first week post-infection may be more useful for early detection of PRRSV infection. These early antibodies include non-neutralizing antibodies specific for structural proteins such as PRRSV N protein or nsps [1, 11]; N protein and certain nonstructural proteins (nsp1, nsp2 and nsp7) have been demonstrated to be highly immunogenic [12, 13]. Indeed, most currently available commercial ELISA kits that detect PRRSV-specific antibodies (e.g., IDEXX HerdChek PRRS ELISA) employ anti-N antibody as a serological marker for PRRSV infection or Modified Live Virus (MLV) immunization status [1].

Although commercial tests such as ELISA are highly sensitive for determining the presence of PRRSV-specific antibodies in serum samples, ELISAs are not suitable for quantitative analysis of antibody levels. This shortcoming is due to the fact that OD values obtained from ELISAs usually vary within a narrow range (from 0.1 to 2). Moreover, most ELISA kits use prokaryotically expressed single recombinant PRRSV structural antigens (generally PRRSV-N protein) as coating antigens. Consequently, such systems cannot evaluate PRRSV-specific antibody responses against other PRRSV enveloped proteins or nonstructural proteins (nsps). It should be noted here that systems employing multiple PRRSV antigens will probably not be developed due to the tremendous effort required for expression and purification of multiple ELISA plate coating antigens. Moreover, due to the general nature of ELISA coating antigens expressed in *E. coli*, false positive or false negative results have been frequently reported [12, 14]. Therefore, development of an improved method for both accurate detection and quantification of PRRSV specific antibodies is urgently needed.

In this study, we developed a modified assay based on luciferase immunoprecipitation systems (LIPS) and hereafter referred to as the luciferase-linked antibody capture assay (LACA) for PRRSV specific antibody detection, [15–17]. Briefly, the LACA detects PRRSV-specific antibodies in pig serum samples using mammalian cell-expressed recombinant PRRSV protein antigens (N and nsp1α) fused with *Rellina* luciferase. Similar to LIPS, LACA utilizes the enzymatic activity from captured luciferase-fused antigen-antibody complexes to convert a substrate to luminescent form to quantify antibody levels indirectly [15, 16]. As an immunoprecipitation assay, LIPS was originally developed by Dr. Peter D. Burbelo from the National Institutes of Health (NIH). LIPS utilizes luciferase-fused antigen and has been used for detection of antigen-specific antibodies or autoantibodies resulting from infection or autoimmune disease, respectively [18–20].

Compared to other antibody detection assays, antigen used for LIPS is easily obtained from cell lysates of mammalian cells previously transfected with plasmids coding for luciferase-fused antigens. Although few reports detail the use of LIPS for swine pathogen, a recent study reported the application of LIPS to the characterization of swine acute diarrhea syndrome coronavirus (SADS-CoV) originating in bats in China [21].

In this study, a modified ELISA-like form of LIPS, the LACA, was adapted as a diagnostic method for detection of PRRSV-specific antibodies. Two antigen targets (PRRSV-N protein and nsp1α) were selected for evaluation of specificity and sensitivity of the LACA for anti-PRRSV antibody detection using serum samples that were either negative or positive for anti-PRRSV antibodies. Moreover, sequentially collected serum samples obtained from piglets infected with PRRSV were evaluated for anti-PRRSV antibodies to compare the sensitivity of LACA to ELISA for earlier detection of anti-PRRSV antibodies during the course of PRRSV infection.

## Methods

### Cells, virus, plasmids, chemical reagents

MARC-145, HEK293T and BHK-21 cells were purchased from the China Center for Type Culture Collection (CCTCC, Wuhan, China) and maintained in Dulbecco’s modified Eagle’s medium (Gibco, Carlsbad, CA, USA) supplemented with 10% fetal bovine serum (FBS) (*v*/v; BI, Israel) at 37 °C and 5% CO_2_. Two highly pathogenic genotype 2 PRRSV isolates, SD16 (GenBank Accession No. JX087437.1) and HuN4 (GenBank Accession No. EF635006.1) were used in this study. Plasmid transfections of HEK293T and BHK-21 cells were conducted using FuGENE-HD transfection reagent (Promega, Madison, WI, USA) in accordance with the manufacturer’s instructions.

The cDNA sequences coding for PRRSV-N protein or PRRSV-nsp1α were cloned from infectious clones of PRRSV-SD16 and HuN4 strains, respectively, using Q5® High-Fidelity DNA Polymerase (New England Biolabs, Ipswich, MA, USA) [22]. The *Renilla* luciferase coding sequence DNA was cloned from pGL4.74-*hRL*-TK plasmid (Promega) and fused with PRRSV-N or PRRSV-nsp1α coding sequences at their C-terminal ends via overlapping PCR that introduced a 10-amino acid (AA)-length linker protein to connect luciferase to antigen. *Renilla* luciferase-linked PRRSV-N or PRRSV-nsp1α were further cloned into *EcoRI* and *NotI* sites of the pCAGEN expression vector (Addgene plasmid #11160). Primers used for molecular cloning are listed in Table 1.

**Table 1:** Primer list for overlapping PCR to construct PRRSV-N-*Rluc* and nsp1α- *Rluc* plasmids

	Forward primer	Reverse primer
SD16 N	CGGAATTCATGCCAAATAACAACGGC	CGGTCCGCTACCGGAGCCGCTTGCTG AGGGTGATGCTGT
HuN4 nsp1α	CAGAATTCATGTCTGGGATACTTG	CGGTCCGCTACCGGAGCCGCTCATAGC ACACTCAAAAGG
*Renilla* luciferase	TCCGGTAGCGGACCGGTCGCCACCCTTCCAAGGTGTAC	CCGCGGCCGCTTACTGCTCGTTCTTCAG

### Animal experiment

The animal experiments for obtaining the sequential serum samples after PRRSV infection were performed according to Chinese Regulations of Laboratory Animals(approval license number: NWAFU 20131017/02) and was approved by the Animal Care and Use Committee of Northwest A&F University. PRRSV-free piglets (28 days old) were obtained from PRRS free farm and randomly divided into 2 groups. Group 1 piglets (*n* = 5) received PRRSV only, Group 2 piglets (*n* = 5) were used as the negative control. The animals were kept in 2 separate rooms and fed a commercial diet and water ad libitum throughout the experiment. The piglets of Group 1 were challenged i.n. with PRRSV HuN4 strain (7.5 × 10^5^ TCID50/pig). Serum samples used for validating the LACA assay were collected from our previous animal experiments [23–25].

### Preparation of *Renilla* luciferase-fused PRRSV-N and PRRSV-nsp1α antigens

HEK293T cells were transfected with *Renilla* luciferase vector pGL4.74 *hRL*-TK (Promega) or plasmids coding for *Renilla* luciferase-fused PRRSV-N or nsp1α. Transfected cells were lysed 48 h later using passive cell lysis buffer (Promega) supplemented with a protease inhibitor cocktail (Sigma-Aldrich). Cell lysates were further clarified by centrifugation at 13000 rpm for 5 min to remove cell debris then supernatants were transferred to fresh tubes and stored at − 80 °C before use in the LACA assay. Meanwhile, Western blot analysis and an immunofluorescence assay (IFA) were used to test expression levels of recombinant fusion proteins.

Luciferase levels within cell lysates were evaluated using *Renilla* luciferase substrate (Transgene Biotech, Beijing, China) according to the manufacturer’s instructions and quantified using a VICTORX™ Multilabel Reader (Perkin-Elmer Life and Analytical Sciences, Wellesley, MA, USA). Recombinant *Renilla* luciferase (Raybiotech, Norcross, GA, USA) was used to determine the relative enzymatic activities of luciferase-fused PRRSV-N or nsp1α from cell lysates. For the LACA, enzyme activity values were assigned to HEK293T cell lysate dilutions in phosphate buffered saline (PBS) (100 μL) based on one enzyme activity equivalent of 1 ng recombinant *Renilla* luciferase defined as 1 Test Unit (1 TU) unless otherwise specified.

### Design of luciferase-linked antibody capture assay (LACA)

(i) Preparation of Protein G-coated plates

Black 96-well polystyrene microplates (Corning Inc., Corning, NY, USA) were used for the LACA assay. Briefly, 1 μg of recombinant Protein G (Smart-Lifesciences, Changzhou, Jiangsu, China) in 100 μL PBS was used to coat polystyrene microplates. Plates were incubated at 4°C overnight. After Protein G coating, unbound Protein G was removed by washing with PBS-T buffer [0.5% (*v*/v) Triton X-100 (Sigma-Aldrich, St. Louis, MO, USA) in PBS]. Next, plates were blocked with PBS-T buffer containing 2.5% gelatin (Sigma-Aldrich) for an additional 12 h and stored at 4 °C until further use.

(ii) Co-incubation of antigen and serum samples

When evaluating pig serum samples using the LACA assay, 20-fold diluted serum samples and PBS-diluted HEK293T cell lysates containing 1 TU *Renilla* luciferase-fused PRRSV-N or PRRSV-nsp1α were mixed together in a final volume of 100 μL. Next, mixtures were incubated in Protein G-coated polystyrene microplates for 2 h at room temperature (RT) followed by eight washes with PBS-T buffer.

(iii) Evaluation of luciferase activity

**Fig. 1:**
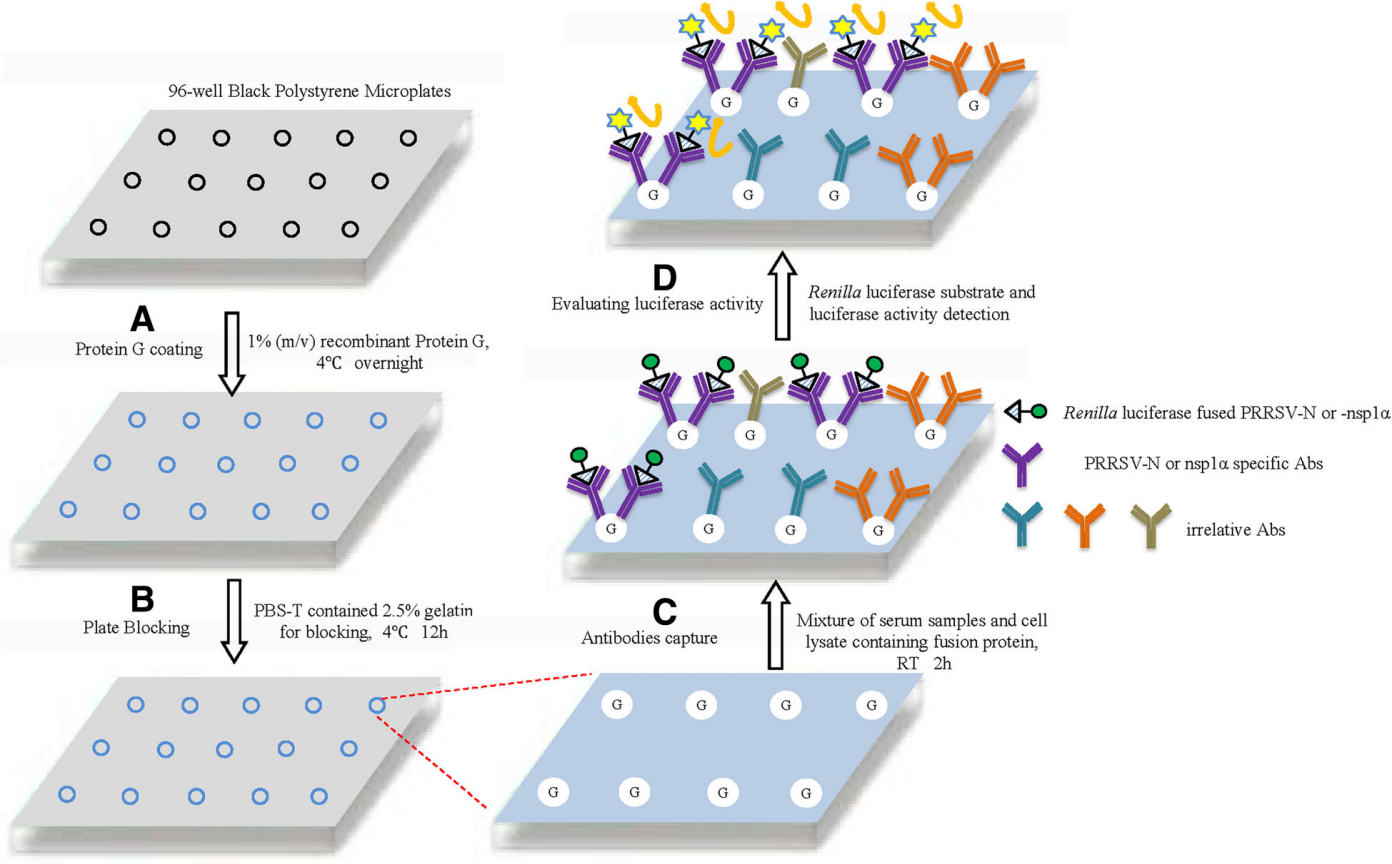
Schematic illustration of luciferase-linked antibody capture assay (LACA); **a** Black 96-well polystyrene microplates were coated with 1 μg of recombinant Protein G; **b** Microplate coated with protein G was blocked by PBS-T buffer containing 2.5% gelatin; **c** The 20-fold diluted serum samples and PBS-diluted HEK293T cell lysates containing 1 TU Renilla luciferase-fused antigen were mixed together and incubated in Protein G-coated well for 2 h at room temperature (RT); **d** Renilla luciferase activity was determined by adding substrate to each well followed by measurement of luciferase activity using a VICTORX™ X5 Multilabel Reader

*Renilla* luciferase substrate was added to each well and plates were incubated for 5 min followed by measurement of luciferase activity using a VICTORX™ ×5 Multilabel Reader. Pig serum samples from specific-pathogen-free (SPF) pigs that were negative for antibodies against PCV-2, PRRSV and porcine epidemic diarrhea virus (PEDV) were included as negative controls. Data were presented as the ratio of luciferase activity of each sample to the luciferase activity of negative serum (*S/N* ratio). The calculation of the *cutoff* value for the *S/N* ratio was determined using values from serum samples previously shown to be positive for antibodies to PRRSV (collected from pigs experimentally infected with PRRSV) as compared to values of negative serum samples from SPF pigs. A schematic illustration of LACA design is shown in Fig. 1.

### Validation of the LACA

(i) Cutoff determination, diagnostic sensitivity and diagnostic specificity

To determine the sensitivity and specificity of the LACA luciferase-linked antigens PRRSV-N and PRRSV-nsp1α (designated N-*luc* LACA and nsp1α-*luc* LACA), 247 serum samples from either PRRSV-infected pigs or SPF pigs were tested. A total of 125 anti-PRRSV antibody-positive samples from pigs experimentally infected with HP-PRRSV strains SD16 (*n* = 32) or HuN4 (*n* = 93) were used as positive controls. All serum samples were collected within the time frame of 10 dpi to 38 dpi post-PRRSV infection. Serum samples (*n* = 122) from PRRSV-free SPF pigs or sera collected prior to experimental PRRSV infection were included as negative controls. All anti-PRRSV antibody-positive serum samples or negative samples were further confirmed by IFA of PRRSV-infected MARC-145 cells. Receiver operating characteristic (ROC) analysis assessments were performed using MedCalc (Version 15.2, MedCalc Software, Belgium).

(ii) (ii) Measurement of repeatability

Repeatability of the LACA was assessed by running the same positive and negative internal control sera. Intra-plate precision was calculated from 20 replicates per plate and inter-plate precision was calculated for each single serum sample across 10 different plate runs. Repeatability was assessed by the coefficient of variation (CV) (CV = SD/Mean). The CV, means and standard deviations (SD) were calculated as previously reported [14]. A CV value of the intra-plate assay of less than 15% was considered to be an acceptable repeatability value.

(iii) Measurement of assay sensitivity using sequential samples

To compare assay sensitivity, 38 sequential serum samples collected at various sequential time points from 6 pigs experimentally infected with PRRSV-HuN4 strain were examined by LACA and IDEXX ELISA at 3–4 days and weekly and results were compared.

(iv) Evaluation of field serum samples

A total of 107 PRRSV-positive (as defined by IDEXX ELISA) field pig serum samples collected from pigs on various farms (supplied by Harbin Veterinary Research Institute) were further tested by both LACA with IFA verification.

### Western blot analysis

Sodium dodecyl sulfate-polyacrylamide gel electrophoresis (SDS-PAGE) and Western blot analysis were conducted as previously described [26, 27] with the following modifications. Briefly, whole cell lysates from plasmid-transfected HEK293T cells were harvested using Laemmli Sample Buffer (Bio-Rad Laboratories, Hercules, CA, USA) then separated using 12% SDS-PAGE and transferred onto PVDF membranes (Millipore, Billerica, MA, USA). Antibodies against *Renilla* luciferase (Thermo Fisher Scientific), β-tubulin (Sigma-Aldrich) and anti-PRRSV-N monoclonal antibody generated in-house (clone No. 6D10) were used for specific protein detection. Detection of primary antibody binding to targets was conducted by incubation of membranes with goat anti-rabbit IgG-conjugated or anti-mouse IgG-conjugated horseradish peroxidase (Sigma-Aldrich) and visualized after addition of ECL chemiluminescence substrate (Bio-Rad Laboratories). The chemiluminescence signal was recorded digitally using a ChemiDoc MP imaging system (Bio-Rad Laboratories). Digital signal acquisition and densitometry analyses were conducted using ImageLab Software, Version 5.1 (Bio-Rad Laboratories).

### Immunofluorescence assay (IFA)

IFA was carried out as reported previously using anti-PRRSV antibody-positive pig serum and rabbit anti-*Renilla* luciferase polyclonal antibody (Thermo Fisher Scientific) [27]. Briefly, BHK-21 cells transfected previously with indicated plasmids were fixed with 4% paraformaldehyde (Sigma-Aldrich) and permeabilized with PBS-T buffer. Specific antibody-protein reactions were detected by either FITC-labeled goat anti-swine IgG conjugates (Jackson ImmunoResearch, West Grove, PA, USA) or DyLight 549 goat anti-rabbit IgG conjugate (Rockland Immunologicals, Gilbertsville, PA, USA). Each coverglass was mounted onto a slide using SlowFade Gold antifade reagent containing 4′6’-diamidino-2-phenylindole (DAPI) (Invitrogen).

IFA using pig serum samples was conducted using MARC-145 cells infected with PRRSV to confirm the existence of PRRSV-specific antibody. Briefly, MARC-145 cells were seeded into wells of 96-well plates (Corning Inc.) at a density of 0.8 × 10^4^ cells/well. After culture for 16–20 h, cells were incubated with PRRSV strain SD16 at a MOI of 0.1 and cultured for additional 24 h. Cells were then fixed with 4% paraformaldehyde (Sigma-Aldrich) and permeabilized with PBS-T buffer. The diluted serum samples (1:100) were added to paired wells (for PRRSV-infected versus uninfected MARC-145 cells) and incubated for 1 h. After three washes, FITC-labeled goat anti-swine IgG conjugate (Jackson ImmunoResearch) was used to detect specific antibody binding. All images were captured and processed using Leica Application Suite X (Version 1.0. Leica Microsystems, Germany).

### Statistical analysis

Statistical analysis was performed using GraphPad Prism version 5.0 (GraphPad Software, San Diego, CA, USA). Repeatability was assessed using the coefficient of variation (CV) (CV = SD/Mean). A CV value for the intra-plate assay of less than 15% was considered an acceptable repeatability level for the assay. For evaluation of sequential serum samples, differences in indicators between samples and controls were assessed using the Student’s *t*-test. A two-tailed *P*-value of less than 0.05 was considered statistically significant.

To determine the optimal *S/N* ratio *cutoff* value that maximizes both diagnostic specificity sensitivity of the assays, MedCalc software (version 15.2) was used for ROC analysis of both LACA to compare histograms of the results obtained. Two-graph ROC plots for N-*Rluc* LACA and nsp1α-*Rluc* LACA were generated.

## Results

### Expression of *Renilla* luciferase-fused PRRSV-N and nsp1α proteins

**Fig. 2:**
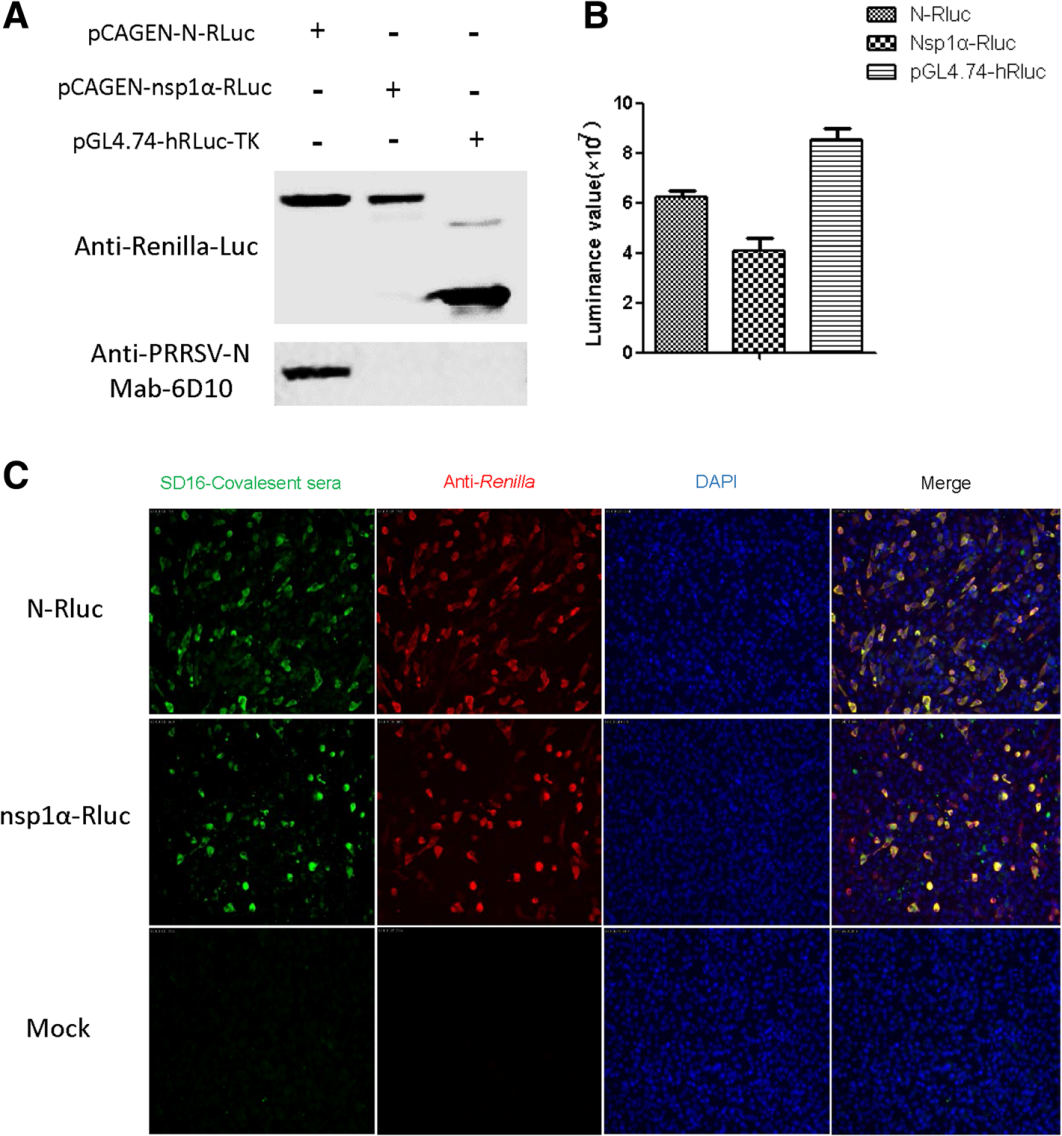
Expression and characterization of *Renilla* luciferase-fused PRRSV-N (N-*Rluc*) and nsp1α (nsp1α-*Rluc*). **a** HEK293T cells were transfected with the indicated plasmids for 48 h followed by detection of expressed recombinant proteins bound to anti-*Renilla* luciferase-conjugated polyclonal antibodies or anti-PRRSV-N monoclonal antibody 6D10 via Western blotting; **b** The luciferase activities of recombinant N-*Rluc* and nsp1α-*Rluc* in cell lysates were evaluated upon addition of *Renilla* luciferase substrate and compared to lysates of cells transfected with pGL4.74-*hRL*-TK. **c** BHK-21 cells transfected with indicated plasmids probed with either PRRSV-SD16 convalescent pig serum (Green channel) or anti-*Renilla* luciferase polyclonal antibodies (Red channel) by immunofluorescence assay

To generate recombinant proteins used for the LACA assay, we first examined the expression of *Renilla* luciferase (*Rluc*)-fused antigens. PRRSV-N-luciferase and NSP1α-luciferase fusion protein expression levels in HEK293T cells were validated using measurements of luciferase activity reflecting the presence of anti-luciferase antibodies. As demonstrated in Fig. 2a, expression levels of both fusion proteins were indirectly measured using anti-*Renilla* luciferase antibody and the results correlated with luciferase activity (Fig. 2b). Meanwhile, monoclonal antibody 6D10 against PRRSV-N protein was used to confirm the expression of *Rlu*-fused N protein (Fig. 2a) and expression of fusion proteins was further confirmed by IFA (Fig. 2c). Based on our data, co-localization of PRRSV-serum positive cells and luciferase positive cells was observed, suggesting that fused PRRSV-N and PRRSV-nsp1α proteins could each be recognized by anti-PRRSV antibody-positive serum in conjunction with *Renilla* luciferase serving as reporter (Fig. 2c).

### Determination of *cutoff* value, diagnostic sensitivity, specificity and repeatability of the LACA assay

**Fig. 3:**
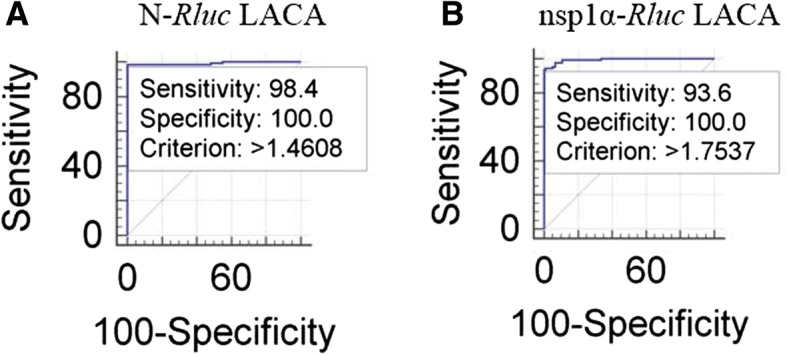
Analysis of the sensitivity and specificity for N-*Rluc* and nsp1α-*Rluc* LACA. **a** Evaluation of sensitivity and Specificity for *N-Rluc* LACA; **b** Evaluation of sensitivity and Specificity for nsp1α-*Rluc* LACA

Serum samples from pigs experimentally infected with PRRSV (SD16 and HuN4 strains) along with 122 serum samples from SPF pigs or sera collected prior to PRRSV infection were further verified by IFA to confirm positivity or negativity for anti-PRRSV antibodies (data not shown). These validated samples were used to determine the *cutoff* value of LACA. An optimized *cutoff* value that maximized assay efficiency was demonstrated for an *S/N* ratio of 1.4608 for N-*Rluc* LACA and an *S/N* ratio of 1.7537 for nsp1α-*Rluc* LACA (Fig. 3a and b, respectively). As shown in Table 2, diagnostic sensitivity rates of 98.4% vs. 93.6% were observed (95% confidence interval, 94.3–99.8% vs. 87.8–97.2%) for N-*Rluc* LACA and nsp1α-*Rluc* LACA, respectively. Moreover, diagnostic specificity rates of 100% vs.100% (95% confidence interval, 97.0–100% vs. 97.0–100%) for N-*Rluc* LACA and nsp1α-*Rluc* LACA were observed as well, indicating that the specificity of N-*Rluc* LACA was comparable to that of nsp1a-*Rluc* LACA (*p* = 0.7870) (Table 3).

**Table 2:** ROC Analysis for N-*Rluc* LACA and nsp1α-*Rluc* LACA

Characteristics	Value for N-*Rluc* LACA	Value for nsp1α-*Rluc* LACA
Optimized cutoff (*S/N*)	1.4608	1.7537
Diagnostic sensitivity (%)	98.4	93.6
95% confidence interval	94.3–99.8	87.8–97.2
Diagnostic specificity (%)	100.0	100.0
95% confidence interval	97.0–100.0	97.0–100.0
AUC	0.992	0.994
95% confidence interval	0.971–0.999	0.974–0.999

**Table 3:** Comparison of N-*Rluc* LACA and nsp1α-*Rluc* LACA

Characteristics	N-*Rluc* LACA vs. nsp1α-*Rluc* LACA
Difference between areas	0.00184
Standard error	0.00680
95% confidence interval	−0.0115 to 0.0152
Significant level	P = 0.7870

With regard to repeatability, N-*Rluc* LACA and nsp1α-*Rluc* LACA were analyzed to determine their potential value for diagnostic applications, while the levels of precision of both LACA systems were compared using antibody-negative internal control sera. The N-*Rluc* LACA intra-plate %CV was 9.79 and the %CV between runs was 6.26, while the nsp1α-*Rluc* LACA intra-plate %CV was 8.72 and the %CV between runs was 8.98 (Table 4).

**Table 4:** Evaluation of Assay repeatability for N-*Rluc* LACA and nsp1α-*Rluc* LACA

Assay	Repeatability result (% CV)	
	Within plate	Between runs
N-*Rluc* LACA	9.79	6.26
nsp1α-*Rluc* LACA	8.72	8.98

### Application of LACA for anti-PRRSV antibody detection in sequential serum samples collected during the course of experimental PRRSV infection

**Fig. 4:**
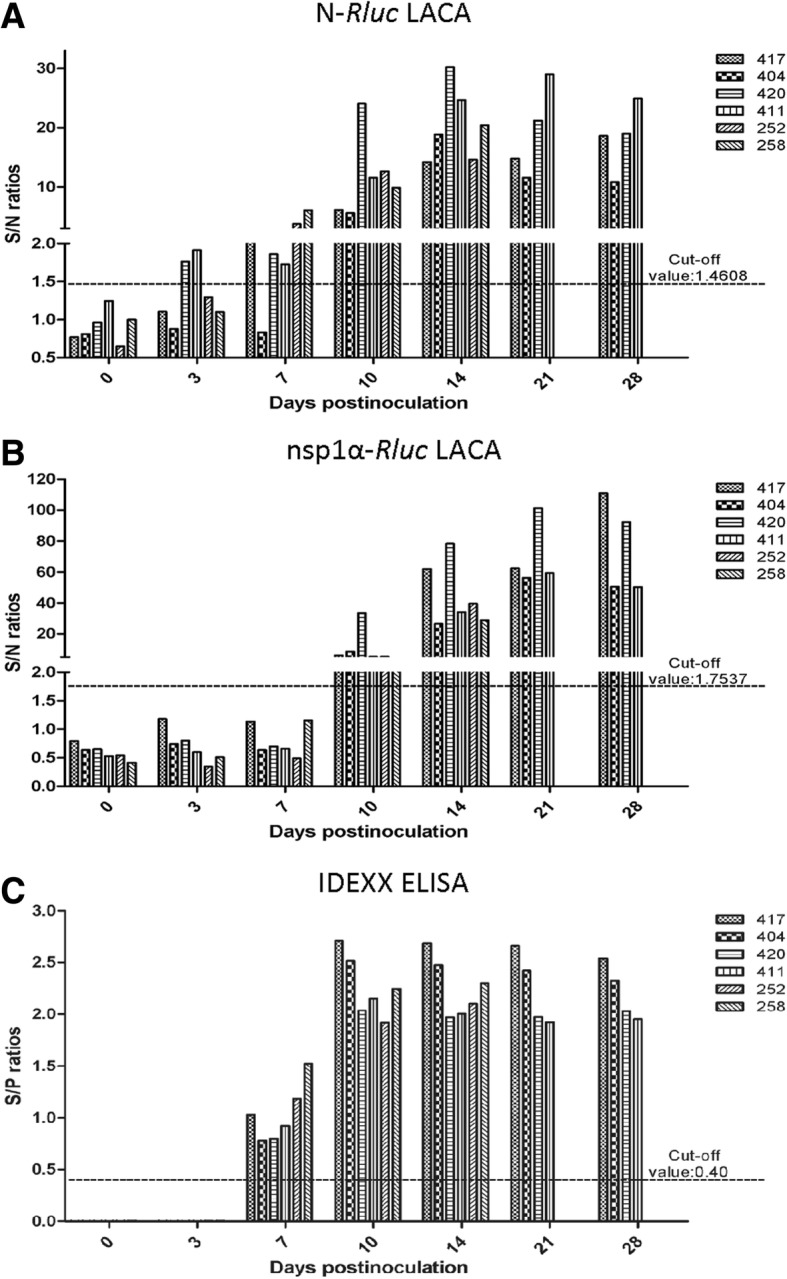
Evaluation of sequential serum samples obtained from experimentally infected pigs by N-*Rluc* LACA, nsp1α-*Rluc* LACA and IDEXX ELISA. Serum samples from 6 pigs experimentally infected with PRRSV-HuN4 strain were collected at indicated time points and evaluated by different methods to compare assay sensitivity between *N-Rluc* LACA (**a**) and nsp1α-*Rluc* LACA (**b**), with the same serum samples tested in parallel using IDEXX ELISA (**c**)

To evaluate the analytical sensitivity of LACA for diagnosis of early PRRSV infection, LACA and IDEXX PRRS ×3 ELISA were performed side-by-side to measure specific antibody detection in 38 serum samples collected at a series of time points from 6 HuN4-inoculated pigs. Among the 6 pigs, sequential samples were collected at 0, 3, 7, 10 14, 21 and 28 dpi and for 4 pigs sequential samples were collected at 0, 3, 7, 10 and 14 dpi (Fig. 4a). Based on our results of the N-*Rluc* LACA, serum samples from two infected pigs (33.33%) were identified as anti-PRRSV antibody-positive as early as 3 dpi and serum samples from five pigs (83.33%) were positive at 7 dpi. All sera collected at 10 dpi were positive using N-*Rluc* LACA (Fig. 4a). For the nsp1α-*Rluc* LACA, PRRSV-specific antibodies were not detected before 7 dpi. However, all serum samples tested positive at 10 dpi and thereafter (Fig. 4b). Meanwhile, all serum samples analyzed by IDEXX ELISA were anti-PRRSV antibody-negative at 3 dpi but tested antibody-positive at 7 dpi (Fig. 4c). Taken together, it appears that the N-*Rluc* LACA demonstrated superior sensitivity for early PRRSV detection (3 dpi) when compared to nsp1-*Rluc* LACA and IDEXX ELISA.

### Evaluation of PRRSV-specific antibodies in field serum samples using the LACA

Next, both N-*Rluc* LACA and nsp1α-*Rluc* LACA were evaluated for detection of PRRSV-specific antibodies in sera collected from farmed pigs infected with unknown field PRRSV strains. To perform this comparison, a total of 107 PRRSV-positive (as defined by IDEXX ELISA) field serum samples collected from pigs from various farms were further tested by both LACA assays along with IFA verification. Among these field samples, an IDEXX ELISA-positive serum sample was found to be negative using both IFA and LACA (both N and nsp1α), suggesting a false-positive result for this sample based on IDEXX ELISA. In comparison with the results from IFA, 102 of 106 (96.2%) IFA-positive samples were defined as anti-PRRSV antibody-positive by N-*Rluc* LACA, while 101 of 106 IFA-positive samples (95.3%) were defined as positive by nsp1α-*Rluc* LACA (Table 5). Among unexpected false-negative results generated by both LACA tests, 2 of these results were associated with the same serum samples.

**Table 5:** Comparison of field samples detected by LACA, IDEXX ELISA and IFA

Serum Group	No. of seropositive detected by analysis/total No. of tested serum samples		
	N-*Rluc* LACA	nsp1α-*Rluc* LACA	IFA
IDEXX ELISA-positive	102/107	101/107	106/107

## Discussion

In this study we developed an assay, the luciferase-linked antibody capture assay (LACA), and conducted a proof-of-concept validation of LACA for use in detection of PRRSV-specific antibodies in pig serum samples. There is another assay utilizing luciferase-linked antigen for antibody detection, the luciferase immunoprecipitation systems (LIPS) assay [16]. LIPS uses immobilized Protein A/Protein G beads to pull down antigen-antibody complexes for antigen-specific antibody detection and quantification [15, 16, 28]. It has been widely used for pathogen-specific antibody detection including swine pathogen porcine circovirus-2 (PCV-2) [29], as well as for auto-antibody profiling of autoimmune disease patients [20, 30–32]. However, compared to LACA, there are certain disadvantages for LIPS while both assays employ luciferase-fused antigen and luminance signal from luciferase substrate for indirect detection and quantification of antigen-specific antibodies. On the one hand, the LIPS assay requires skilled technicians to conducted complicated immunoprecipitation (IP) procedures. On the other hand, LIPS requires immobilized protein G or A beads (agarose or magnetic beads conjugated to Protein G or A) which are less cost-effective for large scale screening beyond academic research.

As demonstrated in Fig. 1, LACA is a protocol that shares major steps with ELISA, including incubation and well washes, with comparable total times for both assays. However, the two types of assay differ in reporter enzymes used (HRP for ELISA, luciferase for LACA), substrates (TMB for ELISA, luciferin for LACA), antigens (plate-coated antigen for ELISA, cell lysates for LACA) and opacity of plates (transparent for ELISA, black for LACA). Advantages of LACA over ELISA include the fact that luciferase-linked viral antigens used in LACA can be directly obtained from lysates of plasmid-transfected cells without requiring purification procedures [33–36]. Moreover, luciferase activity in LACA generates highly quantitative data spanning a relatively wider range of values (from 10^3^ to 10^6^) than for ELISA, which is more suitable for evaluating dynamic changes in antibody responses against a specific antigen over time.

PRRSV-N protein is a highly immunogenic protein and evokes a rapid humoral immune response after PRRSV infection [1]; therefore, PRRSV-N specific antibody production has been widely accepted as a serological marker for PRRSV infection or vaccine immunization status. In our study, the PRRSV-specific antibody response could be measured as early as 3 dpi by N-*Rluc* LACA, but not by IDEXX ELISA for the same panel of sequential serum samples. These results therefore indicate greater analytical sensitivity of the LACA than of ELISA, perhaps due to LACA detection of swine anti-N IgM; because no N-specific IgG would be expected to be produced by 3 dpi, it is possible that the Protein G coating used in the LACA could capture N-specific IgM or other N-specific antibody types from swine sera. Nevertheless, the maximal level of antibody detected at 14 dpi by N-*Rluc* LACA was quite similar to that analyzed by IDEXX ELISA. As noted a decade ago, humoral immune responses against PRRSV infection are not restricted to PRRSV structural proteins, since antibodies recognizing nsps were detected as early as one week after PRRSV infection [1] with certain nonstructural proteins (nsp1, nsp2 and nsp7) highly immunogenic [13]. Since nsp1α is both the first nsp produced post-infection and the first viral protein synthesized during PRRSV replication in cells, we also tested *Renilla* luciferase-fused nsp1α as an alternative antigen for LACA. Based on our results, the earliest detection of PRRSV-specific antibody was observed at 10 dpi for nsp1α-*Rluc* LACA, with a longer duration of antibody detection that peaked at 21 dpi and remained high at 28 dpi (the end time point of the experiment).

When compared to well-established commercial ELISA tests for PRRSV-specific antibody detection, false negative results were observed during LACA testing of field serum samples randomly collected from swine herds, suggesting the need for further improvement and optimization. On the one hand, since the luciferase-fused PRRSV antigens used in this study (both N and nsp1α) were each derived from a single PRRSV strain (SD16 for N and HuN4 for nsp1α), it is possible that PRRSV strains circulating within the swine herd possessed corresponding proteins with different antigenic determinants, resulting in false negative results. Moreover, only 125 confirmed positive and 122 confirmed negative serum samples were used to validate the LACA assay, a limited sample size. Therefore, systematic screening for luciferase-fused PRRSV antigen or a combination of different PRRSV antigens may significantly improve LACA sensitivity.

## Conclusion

The LACA is a highly adaptable method for large-scale screening or screening of antibodies to multiple antigenic targets and provides results that are comparable to results obtained using a well-established PRRSV ELISA assay kit. Moreover, the LACA could detect PRRSV infection earlier (at 3 dpi) and with greater sensitivity than detected by ELISA.

## Abbreviations

AA: Amino acid
CSFV: Classical Swine Fever virus
DAPI: 4′6’-diamidino-2-phenylinodole
DIVA: Differentiation of infected and vaccinated animals
dpi: Days post-inoculation
ELISA: Enzyme-linked immunosorbent assay
HRP: Horseradish peroxidase
IFA: Immunofluorescence assay
IP: Immunoprecipitation
IPMA: Immunoperoxidase monolayer assay
LACA: Luciferase-linked antibody capture assay
LIPS: Luciferase Immunoprecipitation Systems
Nabs: Neutralizing antibodies
Non-Nabs: Non-neutralizing antibody
nsp1α: Nonstructural protein 1α
ORF: Open reading frames
PBS: Phosphate buffer saline
PCV2: Porcine Circovirus Type 2
PEDV: Porcine Epidemic Diarrhea Virus
PRRS: Porcine reproductive and respiratory syndrome
PRRSV: Porcine reproductive and respiratory syndrome virus
PTM: Post translation modification
ROC: Receiver operating characteristic
RT: Room temperature
*S/N* ratio: Sample to negative ratio
SD: Standard deviations
SDS-PAGE: Sulfate-polyacrylamide gel electrophoresis
TU: Test Unite
